# Gynaecoid Pelvis among Female Patients Attending Department of Radiology of a Tertiary Care Centre: A Descriptive Cross-sectional Study

**DOI:** 10.31729/jnma.8127

**Published:** 2023-04-30

**Authors:** Bipana Manandhar, Esha Shrestha

**Affiliations:** 1 Department of Anatomy, Kantipur Dental College, Basundhara, Kathmandu, Nepal; 2 Department of Physiology, Kantipur Dental College, Basundhara, Kathmandu, Nepal

**Keywords:** *female*, *pelvis*, *radiology*

## Abstract

**Introduction:**

The bony pelvis consists of the two hip bones, the sacrum and the coccyx. The bony pelvis is divided into the greater pelvis and the lesser pelvis. The junction between the greater and the lesser pelvis is the pelvic inlet. The transverse and anteroposterior dimensions of the pelvic inlet will classify the pelvis as the anthropoid, gynaecoid, android, and platypelloid pelvis. Knowledge of female pelvis type is important for obstetricians to know the process of labour which can decrease the morbidity and mortality of mothers and neonates. Thus, the aim of this study was to find out the prevalence of gynaecoid pelvis among female patients attending the Department of Radiology of a tertiary care centre.

**Methods:**

This was a descriptive cross-sectional study conducted in the Department of Radiology of a tertiary care centre from 24 July 2022 to 15 November 2022 after approval from the Institutional review committee (Reference number: 11/022). The study included radiographs of the female pelvis without any bony pathology and developmental anomalies. Anteroposterior and transverse dimensions of the pelvic inlet were measured using a digital ruler in a computer. A convenience sampling method was done. Point estimate and 95% confidence interval were calculated.

**Results:**

Among total female patients, the gynaecoid pelvis was found in 28 (46.66%) (34.04-59.28, 95% Confidence Interval). Mean anteroposterior and transverse diameters for the gynaecoid pelvis were observed to be 12.85±1.0 cm and 13.66±1.07 cm respectively.

**Conclusions:**

The prevalence of gynaecoid pelvis was similar to the other similar studies conducted in similar settings.

## INTRODUCTION

The pelvis is a basin-shaped structure that holds the vertebral columns and protects the abdominal organs. It is made of sacrum, coccyx and two hip bones. The female bony pelvis is divided into a greater or false pelvis which is above the pelvic brim and a true or lesser pelvis which is below the pelvic brim.^1^ The true pelvis is related to childbearing so its anteroposterior and transverse dimensions are of great importance to the anatomist, gynecologist, and radiographers to determine the type of female pelvis.^1^

Pelvis imaging methods such as conventional radiography is used in categorising female pelvis into different types such as gynaecoid, anthropoid, android and platypelloid pelvis. The knowledge of dimensions and the most prevalent type of pelvis among female patients can avoid complications during delivery.^2^

The aim of this study was to find out the prevalence of gynaecoid pelvis among female patients attending the Department of Radiology of a tertiary care centre.

## METHODS

This descriptive cross-sectional study was conducted on 60 pelvic digital radiographs of female patients aged 25-65 years visiting Department of Radiology of Kantipur Dental College and Teaching Hospital (KDCTH). The study was conducted from 24 July 2022 to 15 November 2022 after obtaining ethical approval from the Institutional review committee (Reference number: 11/022) of KDCTH. Anteroposterior radiographs of the normal female pelvis of good resolution were included. Exclusion criteria included radiographs of the female pelvis with fractured pelvic bone, pelvic bone pathology or any developmental anomalies. Convenience sampling was done. The sample size was calculated by using the following formula:


$$\begin{array}{ll} \text{n} & ={\text{Z}}^{2}\times \frac{\text{p}\times \text{q}}{{\text{e}}^{\text{2}}}\\ & =1.96^{2}\times \frac{0.08\times 0.92}{0.07^{2}}\\ & =58 \end{array}$$

Where,

n = minimum required sample size
Z= 1.96 at 95% Confidence Interval (CI)
p = prevalence of gynaecoid pelvis taken from the previous study as, 8%^3^
q = 1-p
e = margin of error, 7%

The calculated sample size was 58. However, 60 sample size was taken.

The anteroposterior diameter or conjugate diameter of the pelvic inlet was measured as the distance from the superior border of the pubic symphysis to the sacral promontory. The transverse diameter of the pelvic inlet was calculated as the widest distance between iliopectineal lines. The diameters of a radiograph were measured with the use of a digital ruler in a computer. The data collected was analysed by statistical analysis using SPSS 20.0. Pelvis was classified into four types.^4^

Anthropoid pelvis refers to the type with an anteroposterior diameter greater than the transverse diameter.

Gynaecoid pelvis refers to the type with anteroposterior and transverse diameters that are equal or the transverse diameter is more than the anteroposterior diameter by not more than 1 cm.

Android pelvis refers to the type with a transverse diameter is more than the anterior diameter by more than 1 cm but less than 3 cm.

Platypelloid pelvis refers to the type with transverse diameter is more than the anterior diameter by 3 cm or more.

The collected data were entered and analysed in Microsoft Excel Version 2010. Point estimate and 95% CI were calculated.

## RESULTS

Among 60 female patients, the prevalence of gynaecoid pelvis was found to be 28 (46.66%) (34.99-58.33, 95% CI). The mean value of the anteroposterior diameter of the pelvic inlet for the gynaecoid pelvis was found to be 12.85±1.00 cm (Table 1).

**Table 1:** Distribution of anteroposterior and transverse diameters of the pelvic inlet.

	mean±SD	mean±SD
Type of pelvis	Anteroposterior diameter	Transverse diameter
Gynaecoid	12.85±1.00	13.66±1.07

Among 28 patients having gynaecoid pelvis, 6 (21.42%) were in the age group of 25-45 years (Table 2).

**Table 2:** Age-wise distribution of patients with the gynaecoid pelvis (n = 28).

Age (years)	n (%)
25-45	6(21.42)
45-65	22 (78.57)

## DISCUSSION

Size and shape of the pelvis are essential to determine the capacity of childbearing which affects the labour process.^5^ This study presented the gynaecoid pelvis as the most prevalent type of pelvis with 46.66%. The prevalence of the gynaecoid pelvis in this study was found nearly equal to the study conducted on Japanese women with 43.6%.^6^ Reduction in the size of the pelvis can lead to morbidity and mortality of the mother and foetus.^7^ Variation in pelvic size and shape could be due to daily activities leading to alteration in body mass. With the advancement of age, ilium becomes less straight in females leading to an alteration in its morphology. Obstetrical dimensions of the female pelvis may get reduced after post-menopause due to a fall in oestrogen levels.^8^ Our study had a slightly higher gynaecoid pelvis (46.66%) than the study carried out in the Department of Radiology, Gailezers Hospital, Latvi in which 40% of the female pelvis showed gynaecoid type. The mean anteroposterior and transverse dimensions of the pelvic inlet of this study were 13.0±0.8 cm and 14.0±0.7 cm respectively for the gynaecoid pelvis. Our study had a slightly lower mean value for both the anteroposterior (12.85±1.0 cm) and transverse dimensions (13.66±1.07 cm).^9^ Our study showed a slightly lower percentage of gynaecoid pelvis than the study conducted in the Department of Radiology, Cumhuriyet University, on multiparous women which resulted in 47.8% of the gynaecoid pelvis. Anteroposterior and transverse diameters were 11. 64±8.0 cm and 14.04±5.7 cm respectively.^10^ Our study had higher mean values for these dimensions.

Disproportion in size of the pelvis of the mother and the head of the foetus can lead to high chances of caesarean delivery. The prevalence of the gynaecoid pelvis in this study was found lower than the study carried out in 400 radiographic films in which 90.3% gynaecoid pelvis was observed.^11^

Genetic influence and adaptational changes to the evolutionary processes may cause an alteration in pelvic size and shape. Climatic changes in different geographical areas can also be the predisposing factor for variation in pelvic size.^12^

Prevalence of gynaecoid pelvis in our study (46.66%) was found to be higher than the study carried out in 1619 pelvic X-rays in which 564 (34.8%) were gynaecoid. This study also showed that maternal height correlates with the dimensions of the pelvic inlet.^13^ Reduced size and shape of the pelvis can lead to premature rupture of membrane and abnormal condition of baby leading to caesarean delivery. The study conducted in the Radiology Department of Hazrat-e Rasool Akram university resulted in 50% gynaecoid. Our study showed the prevalence of gynaecoid pelvis (46.66%) slightly lower than this study.^14^ Differences in pelvic dimensions may occur due to an individual's locomotor activities and postural changes. Our study had a prevalence of gynaecoid pelvis lower than the study conducted on Sudanese women with 50.6%. But anteroposterior and transverse diameters were found to be slightly higher than the anteroposterior (11.7±0.2)and transverse diameter (13.5±0.2) of Sudanese women.^15^

Another study conducted include 54 subjects of various ages resulted in the most common gynaecoid pelvis as 52%.^16^ Our study showed a little lower percentage of prevalence (46.66%) than the above study. The posture of an individual before 14 months of age and strenuous activities performed in adolescence period are also factors influencing the type of pelvis.^17^ In a study conducted on South-South Nigerian females, the prevalence of gynaecoid pelvis was observed to be 38.22%. Our study had a higher prevalence (46.66%) than the above study. Malnutrition has a certain influence on variation in the size of the pelvis.^18^

The limitations of this study are that the dimensions of the pelvic cavity and pelvic outlet were not measured. Further studies can be performed with a larger number of females. Comparative studies can be conducted by including pelvic images of males along with females. Types of the pelvis can be categorised on the basis of race. Pelvic images from computed tomography can be studied for accurate measurements.

## CONCLUSIONS

The prevalence of gynaecoid pelvis among female patients was similar to the studies done in similar settings. Knowledge of the type of female pelvis is essential for obstetrics judgement and prediction of the chances of caesarean delivery.
